# Blastocyst-Derived Lactic Acid May Regulate S100A6 Expression and Function in Mouse Decidualization via Stimulation of Uterine Epithelial Arachidonic Acid Secretion

**DOI:** 10.3390/cells13030206

**Published:** 2024-01-23

**Authors:** Meng-Yuan Li, Ying Wang, Ying Wu, Xu-Yu Zhao, Zhen-Shan Yang, Bo Li, Si-Ting Chen, Yu-Ying He, Zeng-Ming Yang

**Affiliations:** 1 College of Veterinary Medicine, South China Agricultural University, Guangzhou 510642, China; 17835422435@163.com (M.-Y.L.);; 2 Key Laboratory of Animal Genetics, Breeding and Reproduction in the Plateau Mountain Region, College of Animal Science, Guizhou University, Guiyang 550025, China

**Keywords:** decidualization, implantation, S100A6, RAGE, inflammation

## Abstract

(1) Background: Inflammatory responses are implicated in embryo implantation, decidualization, pregnancy maintenance and labor. Both embryo implantation and decidualization are essential to successful pregnancy in rodents and primates. S100A6 is involved in inflammation, tumor development, apoptosis and calcium homeostasis. S100A6 is strongly expressed in mouse decidua, but the underlying mechanisms of how S100A6 regulates implantation and decidualization are poorly defined. (2) Methods: Mouse endometrial stromal and epithelial cells are isolated from day 4 pseudopregnant mouse uteri. Both immunofluorescence and Western blotting are used to analyze the expression and localization of proteins. The molecular mechanism is verified in vitro by Western blotting and the quantitative polymerase chain reaction. (3) Results: From days 4 to 8 of pregnancy, S100A6 is specifically expressed in mouse subluminal stromal cells. Blastocyst-derived lactic acid induces AA secretion by activating the luminal epithelial p-cPLA2. The epithelial AA induces stromal S100A6 expression through the COX2/PGI2/PPAR δ pathway. Progesterone regulates S100A6 expression through the progesterone receptor (PR). S100A6/RAGE signaling can regulate decidualization via EGFR/ERK1/2 in vitro. (4) Conclusions: S100A6, as an inflammatory mediator, is important for mouse implantation and decidualization.

## 1. Introduction

Embryo implantation and decidualization are essential to rodent and human pregnancy [1]. Embryo implantation involves a pro-inflammatory adhesion reaction in the endometrium [2]. Immune cells are enriched in decidua [3]. A dynamic balance of inflammatory responses is essential for decidualization [2,4]. There are many inflammatory molecules involved in implantation and decidualization, including LIF, PGE2, COX2, PGI2, IL-6, IL-8 and TNF [2,5,6]. At the beginning of embryo implantation, the levels of pro-inflammatory cytokines transiently increase [7]. However, the underlying mechanisms of how inflammatory factors regulate implantation and decidualization are poorly defined.

The prostaglandin family is involved in the inflammatory response [8]. Cytosolic phospholipase A alpha (PLA2α) catalyzes the conversion of membrane phospholipids into arachidonic acid (AA), which serves as the precursor to prostaglandin synthesis [9]. Cyclooxygenase 2 (COX2) is an inducible enzyme that catalyzes prostaglandin synthesis. Either cPLA2a or COX2 deficiency leads to implantation failure [10,11]. Exogenous administration of prostacyclin (PGI2) can rescue local decidualization in COX2-deficient mice [11]. COX2-derived PGI2 plays an important function in implantation and decidualization by activating the nuclear peroxisome proliferator-activated receptor (PPAR δ) [9].

S100A6 belongs to the S100 family of Ca^2+^-binding proteins and is expressed as an 89-amino-acid protein [12,13]. S100A6 is involved in inflammation, tumor development, apoptosis and calcium homeostasis [14,15,16,17]. S100A6 can induce inflammation during sterile liver injury in mice [18]. Smoke exposure stimulates S100A6 production to trigger an inflammatory response [19]. Advanced glycation end products (AGEs) and lipopolysaccharide (LPS) co-treatment promotes the upregulation of inflammatory mediators, such as prostaglandins and S100As in MC3T3 cells [20]. Both prostaglandins and S100A proteins are highly detected in glomus tumors [21]. Recent evidence indicates that ATP, uric acid and high mobility group box 1 (HMGB1) in sterile inflammation play important roles during mammalian implantation and decidualization [22,23,24]. Because S100A6 is strongly expressed in mouse decidua [25], we are wondered whether S100A6 is involved in regulating decidualization as an inflammation factor.

In our study, S100A6 is highly expressed in mouse decidual cells. Blastocyst-derived lactic acid stimulates stromal S100A6 expression through the activation of luminal epithelial AA secretion. S100A6 participates in mouse decidualization through the RAGE-EGFR-ERK1/2 pathway.

## 2. Materials and Methods

Animal treatment. CD1 mice (6–8 weeks old) were maintained in a controlled environment (14 h light) and allowed free access to regular food and water. All mouse procedures were approved by the Institutional Animal Care and Use Committee of South China Agricultural University. Fertile or vasectomized mice were mated with female mice to achieve pregnancy or pseudopregnancy. Thirty-two pregnant mice were randomly divided into eight groups. The day of the vaginal plug was defined as day 1 of pregnancy. From days 1 to 4, embryos were flushed from the oviducts or uteri to confirm pregnancy. Successful pregnancy on days 5 and 6 was identified when a blue-bed response was visualized by tail-vein injection of 1% Chicago blue dye (C8679, Sigma-Aldrich, St. Louis, MO, USA). Pregnant uteri were collected and frozen at −80 °C for further analysis.

Twenty-eight adult female mice were randomly divided into seven groups. To analyze the actions of steroid hormones, ovariectomized mice were subcutaneously injected with progesterone (1 mg/mouse, E1024, Sigma-Aldrich) or estrodiol-17β (100 ng/mouse, E8875, Sigma-Aldrich). Control mice were treated with sesame oil (0.1 mL per mouse, Sigma-Aldrich). To investigate whether S100A6 expression is progesterone-receptor-dependent, the progesterone-receptor antagonist RU486 (M8046, Sigma-Aldrich) was injected 1 h before progesterone injection. To examine whether S100A6 is regulated by the estrogen receptor, ICI 182780 (0.5 mg/mouse, V900926, Sigma-Aldrich), an estrogen-receptor antagonist, was injected 30 min before estradiol-17β injection. In order to further explore whether ERα or ERβ is also involved in the expression of S100A6, ovariectomized mice were treated with the Erα-agonist propyl pyrazole triol (PPT, 250 ng/mouse, H6036, Sigma-Aldrich) or the Erβ-agonist diary propionitrile (DPN, 100 ng/mouse, H5915, Sigma-Aldrich). Ovariectomized ERα knock-out (ERα KO) mice were treated with estrogen to verify if S100A6 was regulated by ERα. The uteri were collected at 24 h after subcutaneous injection and frozen at −80 °C for further studies.

Delayed implantation and activation model. Ten pregnant mice were randomly divided into two groups. On day 4 of pregnancy, mice were ovariectomized at 8:30–9:00 and treated with progesterone (1 mg/mouse) from days 5 to 7 to maintain delayed implantation. Delayed implantation was activated by injecting estradiol-17β (25 ng/mouse, 850454, Sigma-Aldrich) on day 7.

Isolation and culture of endometrial stromal cells. Day 4 pseudopregnant mice were used to isolate endometrial stromal cells as previously described, with modification [26]. Briefly, uteri on day 4 of pregnancy were opened longitudinally and digested in HBSS (H4891, Sigma-Aldrich) containing 1% trypsin (0458, Amresco, Cleveland, OH, USA) and 6 mg/mL dispase II (0494207801, Roche Applied Science, Basel, Switzerland) at 4 °C for 1 h, 24 °C for 1 h, and 37 °C for 10 min. After the epithelial cells were separated from the uterus, the remaining uterine tissues were further treated with 0.15 mg/mL collagenase I (17100-017, Invitrogen, Houston, TX, USA) in HBSS. Endometrial stromal cells were seeded into a 12-well plate and cultured in DMEM/F12 (D2906, Sigma-Aldrich) with 10% charcoal-treated FBS (cFBS, 040011A, Biological Industries, Cromwell, CT, USA).

Endometrial stromal cells were induced to undergo in vitro decidualization by 1 µM progesterone and 10 M estradiol-17β in DMEM/F12 containing 2% cFBS. During the in vitro decidualization, endometrial stromal cells were treated with U0126 (ERK inhibitor), FPS-ZM1 (RAGE inhibitor) or an EGFR inhibitor. Additionally, stromal cells were treated with 20 µM AA (181198, Sigma-Aldrich), 20 µM Iloprost (ZK36374, Med Chem Express, Monmouth Junction, NJ, USA), 20 µM GW501516 (Cayman Chemical Company, Ann Arbor, MI, USA), 10 µM NS398 (Med Chem Express), 10 µM GSK0660 (Selleck, Shanghai, China) or the combination of AA and NS398, Iloprost and GSK0660, AA and GSK0660, or GSK0660 and GW501516 for 3 h.

Isolation and culture of mouse endometrial epithelial cells. Day 4 pseudopregnant mice were used to isolate endometrial epithelial cells according to the previous method [27]. In brief, uteri of day 4 pseudopregnant mice were split longitudinally and incubated with 0.3% trypsin and 6 mg/mL dispase II in HBSS for 1.5 h at 4 °C, 30 min at 24 °C and 10 min at 37 °C. The mouse endometrial luminal epithelial cells were obtained by modestly rinsing the uterine tissues in HBSS, and these were seeded into 12-well plates with DMEM/F-12 medium including 10% FBS. The epithelial cells were then treated with lactate (L118493, Aladdin, Shanghai, China) for 3 h.

Coculture of mouse endometrial epithelial and stromal cells. The coculture was performed as previously described [23]. After cultured epithelial cells reached the appropriate confluence, they were treated with 20 mM lactic acid, a combination of 20 mM lactic acid and 10 µM AZD3965 (a MCT-1 inhibitor for blocking lactic acid intake, HY12750, Med Chem Express) or AZD3965 for 3 h, and the conditioned medium was collected and centrifuged to remove cellular debris. The cultured stromal cells were treated with the conditioned media from cultured epithelial cells for 3 h and collected for subsequent analysis.

siRNA transfection. The mouse S100A6 siRNAs and negative control (NC) were purchased from Ribobio Co., Ltd. (Guangzhou, China) and transfected using the Lipofectamine 2000 kit (11668019, Invitrogen).

S100A6 overexpression. The mouse pcDNA3.1-S100A6 and pcDNA3.1 (+) vector were designed and synthesized by Hunan Fenghui Biotechnology (Changsha, China) and transfected using the Lipofectamine 2000 kit. At 24 h after transfection, cells were collected for further analysis.

In situ hybridization. Total RNA was extracted from the day 8 pregnant mouse uterus, reverse-transcribed and amplified with specific mouse S100A6 primers (Sense: GACAGTCCAGGCAACAGG and antisense: CCCAGGAAGGCGACATAC) using the polymerase chain reaction. The amplified fragments were inserted into the pGEM-T plasmid (A1360, Promega Corporation, Fitchburg, WI, USA), and verified via sequencing. To prepare the templates for labeling antisense and sense probes, the recombinant plasmids were amplified with primers for T7 and SP6. Digoxigenin-labeled antisense or sense complementary RNA (cRNA) probes were transcribed according to the digoxigenin RNA-labeling kit (11175025910, Roche Applied Science, Basel, Switzerland).

In situ hybridization was executed according to a previously described method [24]. In brief, 10 μm frozen sections were dried on a hot plate and fixed for 1 h with 4% paraformaldehyde/PBS (P6148, Sigma-Aldrich). After hybridization with the S100A6 probe for 16 h in hybridization buffer at 55 °C, the sections were incubated with the anti-digoxigenin antibody coupled with alkaline phosphatase. The digoxigenin-conjugated sense probe served as the negative control. Endogenous alkaline phosphatase activity was blocked with 2 mM levamisole (1359302, Sigma-Aldrich). Sections were incubated in buffer containing 0.4 mM 5-bromo-4-chloro-3-indolyl phosphate (B6274, Sigma-Aldrich) and 0.4 mM nitro blue tetrazolium (N5514, Sigma-Aldrich) for 30 min. The sections were counterstained with 1% methyl green (M8884, Sigma-Aldrich).

Real-Time Quantitative Polymerase Chain Reaction. Real-time PCR was performed according to the previously described method [28]. Total RNAs were extracted by the TRIzol reagent (9109, Takara, Kusatsu, Japan) and reverse-transcribed into cDNA by the HiScript II Q RT SuperMix (R222-01-AB, Vazyme, Nanjing, China). RT-PCR was implemented with a SYBR Premix Ex Taq kit (Q311-02-AA, Vazyme). The results were calculated by the 2^−ΔΔCT^ method and normalized to RPL7. Primer sequences mentioned in this work are as follows: S100A6 (sense: TGGATGATCTGGACCGTA and antisense ACCCACCACTGGATTTGA); RPL7 (sense: GCAGATGTACCGCACTGAGATTC and antisense ACCTTTGGGCTTACTCCATTGATA); Prl8a2 (sense: AGCCAGAAATCACTGCCACT and antisense TGATCCATGCACCCATAAAA).

Immunofluorescence. Immunofluorescence was performed according to the previously described method [29]. Briefly, frozen sections (10 μm) were dried on a hot plate and fixed with 4% PFA solution for 30 min and treated with 0.1% Triton X-100/PBS. The sections were treated with anti-S100A6 antibody (1:500, ab181975, Abcam, Cambridge, UK) or anti-RAGE antibody (1:500, ab216329, Abmart, Shanghai, China) for 16 h in 4 °C after the sections had been blocked with 10% horse serum for 1 h at 37 °C. After washing three times in PBS, the sections were treated with goat anti-rabbit antibody conjugated with Alexa 488 (G21234, Invitrogen) for 30 min at 37 °C. The section was then counterstained with propidium iodide (P4170, Sigma-Aldrich) and mounted with an antifading mounting medium (Sino Biological, Beijing, China). The fluorescence was observed using a Leica laser-confocal microscope.

Western Blotting. Western blotting was performed according to the previously described method [30]. Briefly, uterine tissues or cultured cells were collected in lysis buffer (50 mM Tris-HCl, pH 7.5; 150 mM NaCl; 0.25% sodium deoxycholate and 1% Triton X-100). The protein concentrations of the samples were quantified with a BCA kit (Thermo Fisher Scientific, Waltham, MA, USA). Protein samples were separated by 12% SDS–polyacrylamide gel electrophoresis (SDS/PAGE) and transferred onto polyvinylidene difluoride (PVDF) membranes (Merck KGaA, Darmstadt, Germany). After blocking in 5% nonfat milk (BBI Life Sciences, Shanghai, China) for 1 h at 25 °C, membranes were incubated for 16 h at 4 °C with anti-S100A6 (1:1000, 1316S, Cell Signaling Technology, Danvers, MA, USA), anti-α-tubulin (1:1000, 2144S, Cell Signaling Technology), anti-RAGE (1:1000, TA5309S, Abamrt, Shanghai, China), anti-p-EGFR (1:1000, 3777s, Cell Signaling Technology), anti-EGFR (1:1000, ab52894 Abcam), anti-P-ERK1/2 (1:1000, 4370s, Cell Signaling Technology), anti-ERK1/2 (1:1000, 9102, Cell Signaling Technology), anti-p-CPLA2 (1:1000, 2831s, Cell Signaling Technology), anti-PPAR δ (1:1000, sc74517, Santa Cruz Biotechnology, Dallas, TX, USA), anti-COX2 (1:1000, 4842s, Cell Signaling Technology), anti-PGIS (1:1000, ab23668, Abcam), anti-SNAIL (1:1000, 3879s, Cell Signaling Technology), anti-E-cadherin (1:1000, 3195s, Cell Signaling Technology), or anti-ZO1 (1:1000, ab221547, Abcam) antibodies. Then, the membranes were treated with the appropriate HRP-conjugated secondary antibody (1:5000, Invitrogen) for 1 h at 25 °C. The signals were visualized with an ECL chemiluminescent kit (Millipore, Burlington, MA, USA) by employing the 5200 Tanon Imaging System.

Enzyme-linked immunosorbent assay. After the cultured medium was collected, cellular debris was removed by centrifuging at 1000× *g* for 5 min. AA concentration was measured with the E-EL-0051c assay kit (Elabscience, Wuhan, China).

Statistical analysis. All of the above experiments were independently repeated at least three times. The data are presented as the mean ± standard deviation (SD). Differences between the groups were measured by using the Student’s *t* test. One-way ANOVA tests were used to evaluate multiple comparisons. A value of *p* < 0.05 was defined as being statistically significant.

## 3. Results

### 3.1. S100A6 Expression in Early Pregnant Mouse Uteri

**Figure 1:**
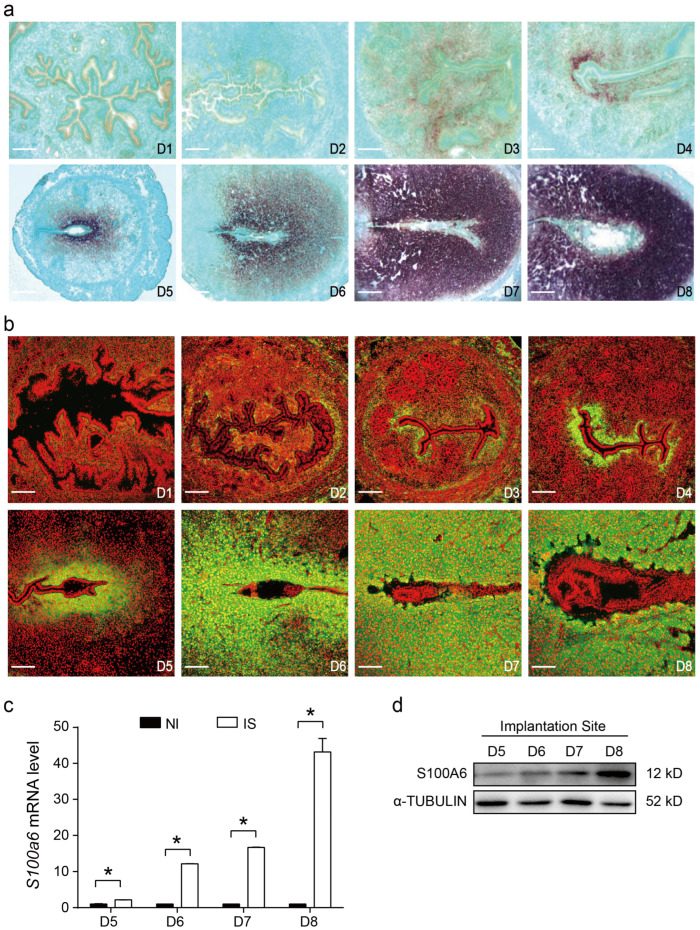
S100A6 expression in early pregnant mouse uteri. (**a**) *S100A6* mRNA in situ hybridization from days 1 to 8 of pregnancy. (**b**) Immunofluorescence of S100A6 protein (green) and propidium iodide (PI; red) from days 1 to 8 of pregnancy. (**c**) RT-qPCR results of *S100A6* mRNA levels at implantation sites (IS) and inter-implantation sites (NI) from days 5 to 8 of pregnancy. (**d**) Western blot results of S100A6 protein levels in mouse uteri at implantation sites (IS) from days 5 to 8 of pregnancy. Scale bar = 250 μm. D1-D8, days 1 to 8 of pregnancy. Bars represent mean ± SD; * *p* < 0.05.

During early pregnancy, *S100A6* mRNA signals first appeared in the subluminal stroma on days 3 and 4 and progressively increased in the subluminal stromal cells around the implanting blastocyst from days 5 to 8 (Figure 1a). The pattern of S100A6 immunofluorescence was similar to that observed by in situ hybridization (Figure 1b). Based on RT-qPCR analysis, *S100A6* mRNA levels from days 5 to 8 of pregnancy were significantly higher at the implantation site compared with inter-implantation sites (Figure 1c). Western blotting also indicated that S100A6 protein levels at implantation sites progressively increased from days 5 to 8 of pregnancy (Figure 1d).

### 3.2. Active Blastocyst Induces S100A6 Expression

**Figure 2:**
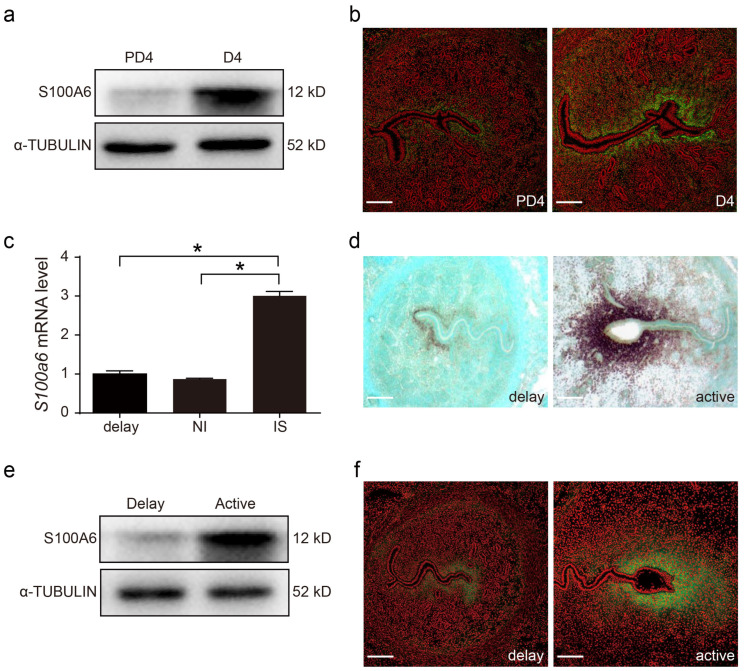
Active blastocysts induce S100A6 expression. (**a**) Western blot results of S100A6 protein levels in day 4 pseudopregnant (PD4) and pregnant uteri (D4). (**b**) Immunofluorescence of S100A6 protein (green) and propidium iodide (PI; red) in day 4 pseudopregnant (PD4) and pregnant uteri (D4). (**c**) RT-qPCR results of uterine *S100A6* mRNA levels undergoing delayed implantation and at implantation sites (IS) and inter-implantation sites (NI) of activated uteri. (**d**) In situ hybridization showing S100A6 expression undergoing delayed implantation and at the implantation site of an activated uterus. (**e**) Western blot analysis of S100A6 protein levels under delayed implantation and at implantation sites (IS) of activated uteri. (**f**) Immunofluorescence of S100A6 protein (green) and propidium iodide (PI, red) undergoing delayed implantation and at the implantation site of an activated uterus. Scale bar = 250 μm. Bars represent mean ± SD; * *p* < 0.05.

Compared with day 4 of pseudopregnancy, uterine S100A6 protein levels on day 4 of pregnancy were higher (Figure 2a). S100A6 immunofluorescence was also stronger on day 4 of pregnancy (Figure 2b). Compared to delayed implantation and inter-implantation sites (NI), the *S100A6* mRNA level at the implantation site (IS) was significantly stronger (Figure 2c). Based on in situ hybridization, the *S100A6* mRNA signal at subluminal stromal cells was stronger after delayed implantation was activated by estrogen (Figure 2d). Western blotting indicated that S100A6 protein signals were also increased in the activated uterus (Figure 2e). Indeed, S100A6 immunostaining also increased at the implantation site of an activated uterus (Figure 2f). These data suggested that blastocysts are involved in regulating S100A6 expression during early pregnancy.

### 3.3. Blastocyst-Derived Lactic Acid Regulation of S100A6 Expression Is Dependent on the AA/COX2/PGI2/PPAR δ Pathway

**Figure 3:**
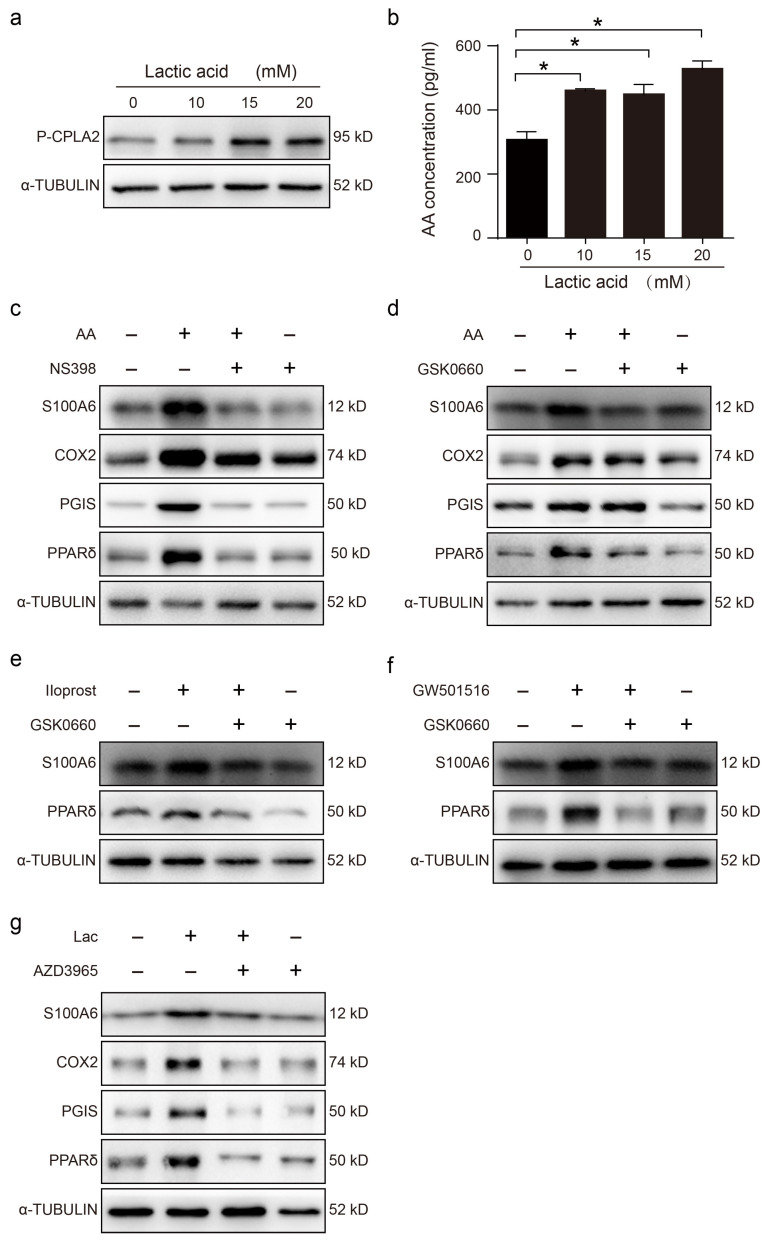
Blastocyst-derived lactic acid regulation of S100A6 expression is dependent on the AA/COX2/PGI2/PPAR δ pathway. (**a**) Western blot showing p-cPLA2 protein level in lactic acid-treated endometrial epithelial cells for 3 h. (**b**) ELISA analysis of the concentration of AA in the epithelial cell-culture medium. (**c**) Western blot showing S100A6, COX2, PGIs and PPAR δ protein levels in endometrial stromal cells treated with AA, AA and NS398, or NS398 for 3 h. (**d**) Western blot analysis of S100A6, COX2, PGIs and PPAR δ protein levels in endometrial stromal cells treated with AA, AA and GSK0660, or GSK0660 for 3 h. (**e**) Western blot analysis of S100A6 and PPAR δ protein levels in endometrial stromal cells treated with Iloprost, Iloprost and GSK0660, or GSK0660 alone for 3 h. (**f**) Western blot showing S100A6 and PPAR δ protein levels in endometrial stromal cells treated with GW501516, GW501516 and GSK0660, or GSK0660 for 3 h. (**g**) Western blot showing S100A6, COX2, PGIs and PPAR δ protein levels in stromal cells after cocultures of uterine epithelial cells and stromal cells were treated with lactic acid, lactic acid and AZD3965, or AZD3965 for 3 h. Lac, lactic acid. Bars represent mean ± SD; * *p* < 0.05.

Lactic acid is secreted by mouse blastocysts and regulates the uterine acidic environment [24,31,32]. When cultured epithelial cells were stimulated with lactic acid for 3 h, the protein level of p-cPLA2 was clearly enhanced (Figure 3a). Similarly, AA secretion was significantly increased (Figure 3b). After stromal cells were stimulated with AA for 3h, the levels of S100A6, COX-2, PGIS and PPAR δ proteins were clearly upregulated, and these were suppressed by NS398, a specific COX2 inhibitor (Figure 3c). AA induction of S100A6 protein levels was also abrogated by GSK0660, a specific PPAR δ antagonist (Figure 3d). The S100A6 protein level was also upregulated by Iloprost, a stable PGI2 analogue, which was abrogated by GSK0660 (Figure 3e). The change in PPAR δ levels was consistent with that of S100A6 (Figure 3e). Furthermore, S100A6 protein levels were also stimulated by GW501516, a PPAR agonist (Figure 3f). When uterine epithelial cells were cocultured with stromal cells, treatment with lactic acid significantly upregulated S100A6, COX-2, PGIS and PPAR δ protein levels, which was abrogated by AZD3965, an MCT-1 inhibitor that blocks lactic acid intake (Figure 3g). These data indicated that blastocyst-derived lactic acid could induce stromal S100A6 expression through an AA-COX2-PGI2-PPAR δ pathway.

### 3.4. Effects of S100A6 on Mouse In Vitro Decidualization

**Figure 4:**
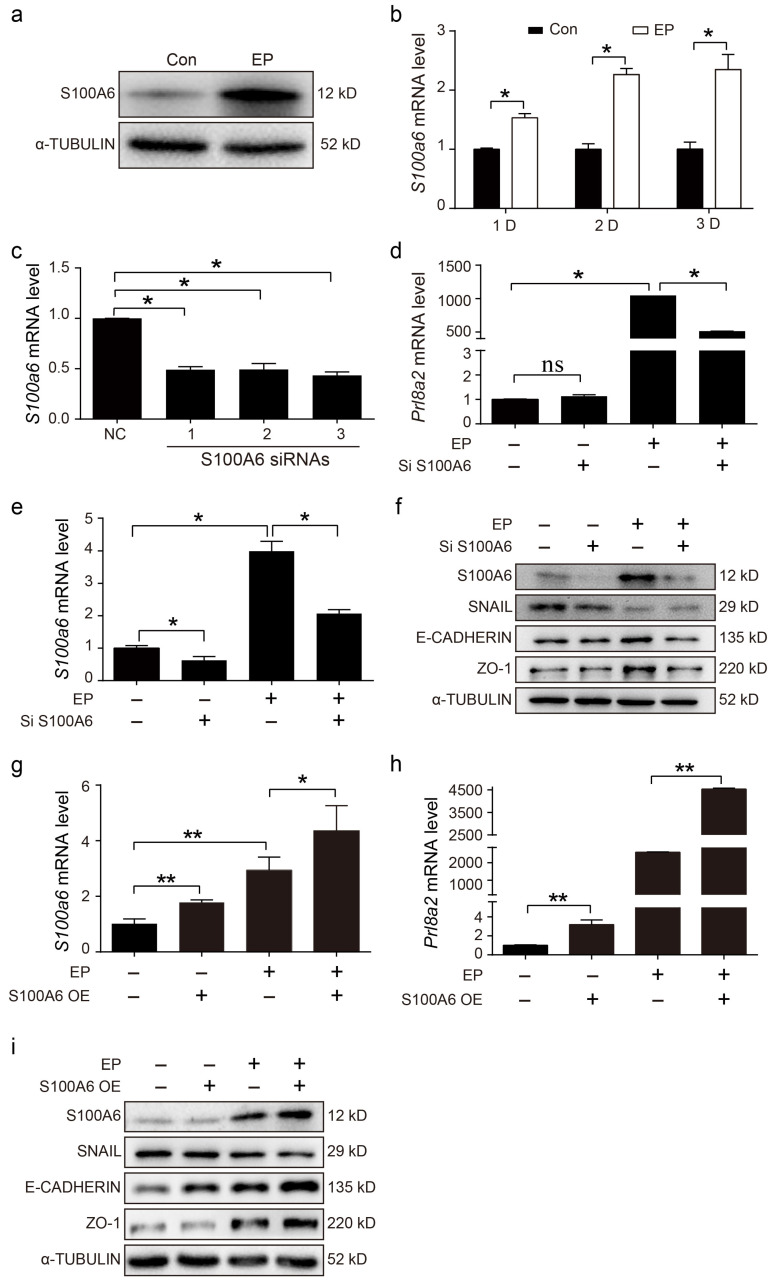
Effects of S100A6 on mouse in vitro decidualization. (**a**) Western blot showing S100A6 protein levels after stromal cells were induced to undergo in vitro decidualization for 48 h. (**b**) RT-qPCR results of *S100A6* mRNA levels during in vitro decidualization from 24 to 72 h. (**c**) RT-qPCR results of the effects of S100A6 siRNAs on *S100A6* mRNA levels in endometrial stromal cells. (**d**) RT-qPCR results of the effects of S100A6 siRNA expression on *Prl8a2* mRNA levels in endometrial stromal cells undergoing in vitro decidualization at 48 h. (**e**) RT-qPCR results of the effects of S100A6 siRNA expression on *S100A6* mRNA levels in endometrial stromal cells undergoing in vitro decidualization at 48 h. (**f**) Western blot results of the effects of S100A6 siRNA expression on S100A6, SNAIL, E-cadherin and ZO-1 protein levels undergoing in vitro decidualization at 48 h. (**g**) RT-qPCR results of the effects of S100A6 overexpression on *S100A6* mRNA levels in endometrial stromal cells undergoing in vitro decidualization at 48 h. (**h**) RT-qPCR analysis of the effects of S100A6 overexpression on *Prl8a2* mRNA levels in endometrial stromal cells under in vitro decidualization at 48 h. (**i**) Western blot results of the effects of S100A6 overexpression on S100A6, SNAIL, E-cadherin and ZO-1 protein levels undergoing in vitro decidualization at 48 h. Si S1006, S100A6 siRNA; S100A6 OE, S100A6 overexpression; EP, estrogen and progesterone treatment. Bars represent mean ± SD; * *p* < 0.05; ** *p* < 0.01; ns, not significant.

Because S100A6 was highly expressed in the decidua zone, in vitro decidualization was performed to examine whether S100A6 might contribute to mouse decidualization in vitro. S100A6 protein levels were dramatically increased after stromal cells were induced for 48 h to stimulate in vitro decidualization (Figure 4a). Compared with the control, *S100A6* mRNA levels were obviously upregulated after stromal cells were induced to undergo in vitro decidualization for 1, 2 and 3 days, respectively (Figure 4b). To further perceive the function of S100A6 during decidualization, S100A6 siRNA and overexpression experiments were performed. Compared with the negative control, *S100A6* mRNA levels were remarkably suppressed by the No.3 siS100A6 sequence (Figure 4c). Upon in vitro decidualization, S100A6 siRNA expression led to a substantial reduction in prolactin family 8, subfamily A, member 2 (Prl8a2) mRNA levels (Figure 4d), a recognized marker for in vitro mouse decidualization [33]. S100A6 siRNA expression also led to a significant reduction in *S100A6* mRNA levels during in vitro decidualization (Figure 4e). In addition, compared with the control, protein levels of E-cadherin and ZO-1 were decreased when S100A6 expression was attenuated during decidualization (Figure 4f). However, the expression of SNAIL showed no obvious change (Figure 4f). S100A6 overexpression significantly upregulated the mRNA levels of *S100A6* under in vitro decidualization conditions (Figure 4g). During in vitro decidualization, S100A6 overexpression significantly upregulated the mRNA levels of *Prl8a2* (Figure 4h). Furthermore, S100A6 overexpression caused an increase in E-Cadherin and ZO-1 protein levels but a decrease in SNAIL levels (Figure 4i). These data show that S100A6 may play a significant role during mouse decidualization.

### 3.5. S100A6 Regulates Decidualization via RAGE

**Figure 5:**
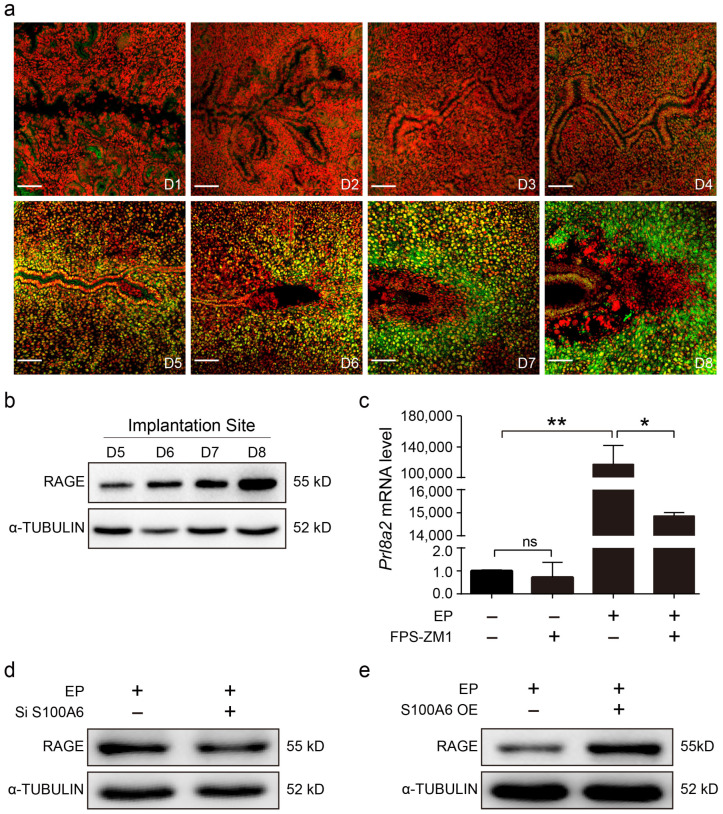
S100A6 regulates decidualization via RAGE. (**a**) RAGE immunofluorescence (green) in mouse uteri from days 1 to 8 of pregnancy. (**b**) Western blot results of RAGE protein levels in mouse uteri at implantation sites (IS) from days 5 to 8 of pregnancy. (**c**) RT-qPCR results of the effects of FPS-ZM1 on Prl8a2 mRNA levels in endometrial stromal cells after in vitro decidualization for 48 h. (**d**) Western blot results of the effects of S100A6 siRNA expression on RAGE protein levels at 48 h of in vitro decidualization. (**e**) Western blot results of the effects of S100A6 overexpression on RAGE protein levels at 48 h of in vitro decidualization. D1–D8, days 1 to 8 of pregnancy. Si S100A6, S100A6 siRNA; S100A6 OE, S100A6 overexpression; EP, estrogen and progesterone treatment. Scale bar = 100 μm. Bars represent mean ± SD; * *p* < 0.05; ** *p* < 0.01; ns, not significant.

Because S100A6 mainly binds the receptor for advanced glycation end products (RAGE) to transduce extracellular effects [34], we examined the pattern of RAGE protein expression in mouse uteri during early pregnancy. From days 1 to 4 of pregnancy, there was weak RAGE immunofluorescence in the stromal cells (Figure 5a). RAGE immunofluorescence gradually increased in the subluminal stromal cells from days 5 to 8 of pregnancy, similar to the S100A6 signal (Figure 5a). Western blotting also showed that RAGE levels at implantation sites progressively enhanced from days 5 to 8 of pregnancy (Figure 5b). FPS-ZM1, an inhibitor of RAGE, significantly inhibited *Prl8a2* mRNA levels during in vitro decidualization (Figure 5c). S100A6 siRNA expression caused a decrease in RAGE protein levels during in vitro decidualization (Figure 5d). Correspondingly, S100A6 overexpression stimulated an increase in RAGE protein levels (Figure 5e). These findings suggested that RAGE might participate in the S100A6 regulation of decidualization.

### 3.6. S100A6 Mediates Decidualization via RAGE/EGFR/ERK1/2

**Figure 6:**
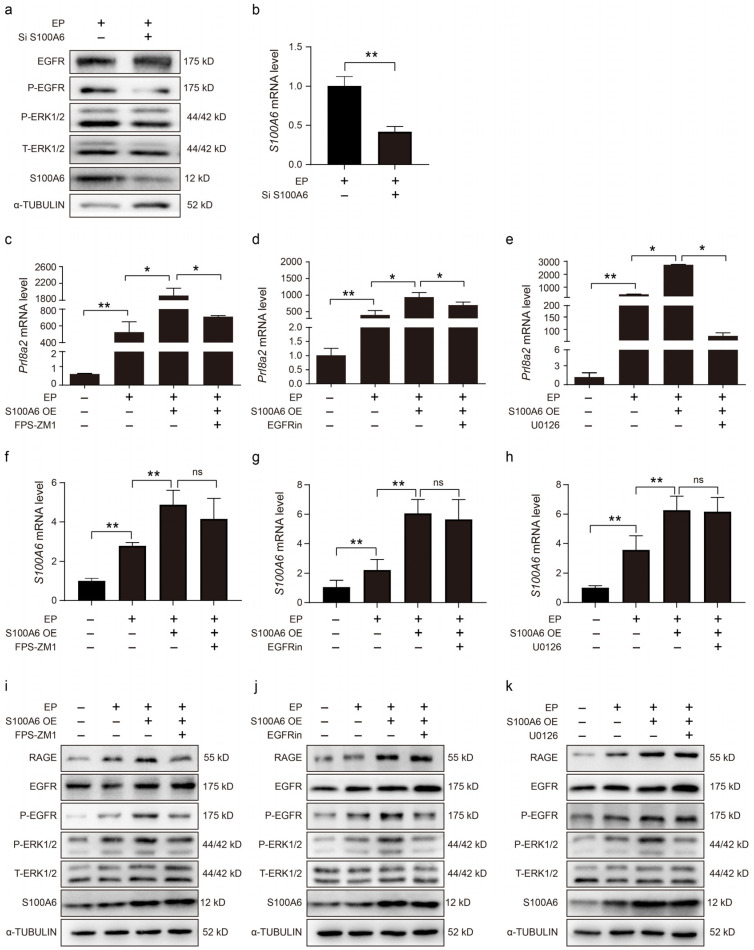
S100A6 mediates decidualization via RAGE/EGFR/ERK1/2. (**a**) The effects of S100A6 siRNA expression on protein levels of EGFR, P-EGFR, T-ERK1/2, P-ERK1/2 and S100A6 in cells undergoing in vitro decidualization at 48 h. (**b**) Effects of S100A6 siRNA on *S100A6* mRNA levels under in vitro decidualization conditions at 48 h. (**c**) Effects of FPS-ZM1 on *Prl8a2* mRNA levels due to S100A6 overexpression under in vitro decidualization conditions at 48 h. (**d**) Effects of an EGFR inhibitor on *Prl8a2* mRNA levels in S100A6-overexpressing cells undergoing in vitro decidualization at 48 h. (**e**) Effects of U0126 on *Prl8a2* mRNA levels in S100A6-overexpressing cells undergoing in vitro decidualization at 48 h. (**f**) Effects of FPS-ZM1 on *S100A6* mRNA levels in S100A6-overexpressing cells undergoing in vitro decidualization at 48 h. (**g**) Effects of an EGFR inhibitor on *S100A6* mRNA levels when S100A6-overexpressing cells at 48 h of in vitro decidualization. (**h**) Effects of U0126 on *S100A6* mRNA levels when S100A6 was overexpressed in cells undergoing in vitro decidualization at 48 h. (**i**) Effects of FPS-ZM1 on RAGE, EGFR, P-EGFR, T-ERK1/2, P-ERK1/2 and S100A6 protein levels in S100A6-overxpressing cells at 48 h of in vitro decidualization. (**j**) Effects of an EGFR inhibitor on RAGE, EGFR, P-EGFR, T-ERK1/2, P-ERK1/2 and S100A6 protein levels when S100A6 was overexpressed cells undergoing in vitro decidualization at 48 h. (**k**) Effect of U0126 on RAGE, EGFR, P-EGFR, T-ERK1/2, P-ERK1/2 and S100A6 protein levels when S100A6 was overexpressed in cells undergoing in vitro decidualization at 48 h. Si S1006, S100A6 siRNA; S100A6 OE, S100A6 overexpression; EGFR in, EGFR inhibitor; EP, estrogen and progesterone treatment. Bars represent mean ± SD; * *p* < 0.05; ** *p* < 0.01; ns, not significant.

Because RAGE can activate ERK1/2 and STAT3 phosphorylation via EGFR [35], we sought to explore the impact of S100A6 on EGFR and ERK1/2 due to in vitro decidualization. S100A6, p-EGFR and p-ERK1/2 protein levels were suppressed by S100A6 siRNA (Figure 6a). In cells undergoing in vitro decidualization, the *S100A6* mRNA level was reduced by S100A6 siRNA (Figure 6b). The increase in *Prl8a2* mRNA levels induced by S100A6 overexpression was suppressed by FPS-ZM1, an inhibitor of RAGE, under conditions of in vitro decidualization (Figure 6c). S100A6-overexpression-induced *Prl8a2* mRNA levels were also suppressed by either an EGFR inhibitor (Figure 6d) or U0126, an inhibitor of ERK (Figure 6e). FPS-ZM1, the EGFR inhibitor, or U0126 had no detectable effects on *S100A6* mRNA levels when S100A6 was overexpressed during in vitro decidualization (Figure 6f–h). Additionally, FPS-ZM1, an inhibitor of RAGE, also inhibited p-EGFR and p-ERK1/2 protein levels during in vitro decidualization when S100A6 was overexpressed (Figure 6i). Both p-EGFR and p-ERK1/2 protein levels were also reduced by the EGFR inhibitor when S100A6 was overexpressed during in vitro decidualization (Figure 6j). Treatment with U0126, an inhibitor of ERK, had no marked effects on p-EGFR and RAGE protein levels when S100A6 was overexpressed (Figure 6k). Collectively, these data suggest that S100A6 may regulate decidualization through RAGE/EGFR/ERK1/2.

### 3.7. Progesterone Regulates S100A6 Expression via the Progesterone Receptor (PR)

**Figure 7:**
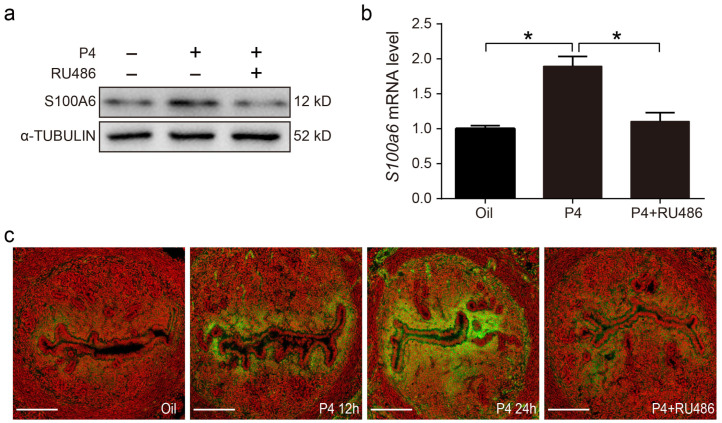
Progesterone regulates S100A6 expression via the PR. (**a**) Western blot analysis of S100A6 protein levels in mouse uteri treated with oil, progesterone, or progesterone and RU486 for 24 h. (**b**) RT-qPCR to detect *S100A6* mRNA levels in mouse uteri treated with oil, progesterone, or progesterone and RU486 for 24 h. (**c**) Immunofluorescence of S100A6 protein expression (green). Scale bar = 100 μm. Bars indicate mean ± SD; * *p* < 0.05.

On day 3 of pregnancy, S100A6 signals were also detected in subluminal stromal cells. Because embryos had not entered the uterine lumen on day 3 of pregnancy, we assumed that rising maternal progesterone from the newly formed corpus luteum may participate in regulating S100A6. In ovariectomized mice, treatment with progesterone for 24 h obviously increased the protein and mRNA levels of S100A6, which was reduced by RU486, a PR antagonist (Figure 7a,b). S100A6 immunofluorescence was also increased by progesterone treatment for 12 and 24 h, which was blocked by RU486 (Figure 7c). These data demonstrate that S1006 expression is controlled by the PR pathway.

### 3.8. Estrogen Regulates S100A6 Expression in an ERα-Dependent Manner

**Figure 8:**
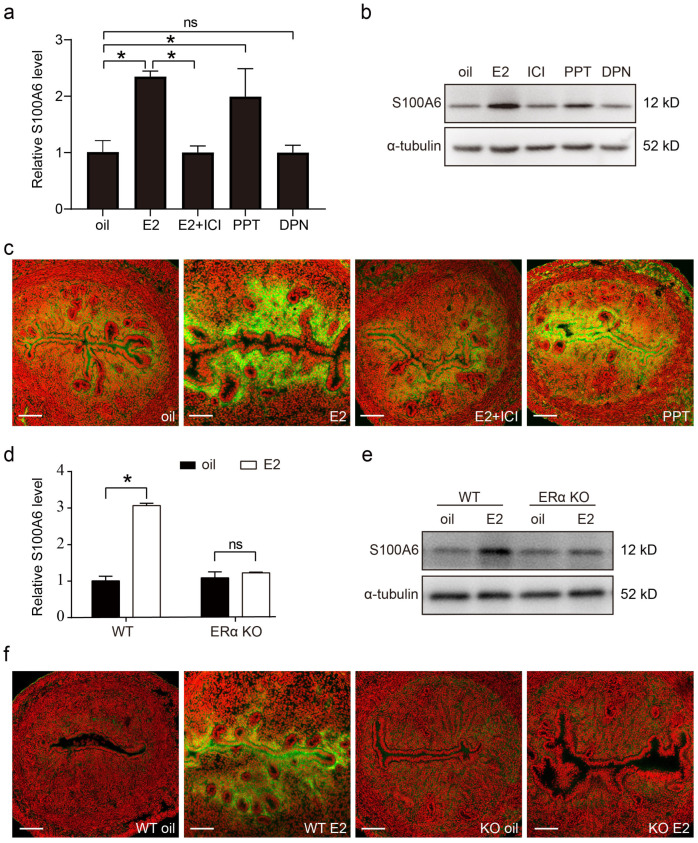
Estrogen regulates S100A6 expression in an ERα-dependent manner. (**a**) RT-qPCR to detect *S100A6* mRNA levels in mouse uteri treated with oil, E2, E2 and ICI, PPT, or DPN for 24 h. (**b**) S100A6 protein levels in mouse uteri treated with oil, E2, E2 and ICI, PPT, or DPN. (**c**) S100A6 immunofluorescence (green) in mouse uteri treated with oil, E2, E2 and ICI, or PPT. (**d**) RT-qPCR to detect *S100A6* mRNA levels in wild type and ERαKO mouse uteri treated with oil or E2 for 24 h. (**e**) S100A6 protein levels in wild type and ERαKO mouse uteri treated with oil or E2 for 24 h. (**f**) S100A6 immunofluorescence in wild type and ERαKO mouse uteri treated with oil or E2 for 24 h. Scale bar = 100 μm. Bars indicate mean ± SD; * *p* < 0.05; ns, not significant.

Estrogen (E2) and progesterone (P4) signaling is finely regulated during embryo implantation and decidualization. In ovariectomized mice, both uterine S100A6 mRNA and protein levels were increased by estrogen treatment, which was counteracted by the estrogen receptor antagonist ICI 182780 (Figure 8a,b). Estrogen action is mainly mediated by ERα and ERβ. PPT, an ERα agonist, also significantly upregulated the levels of S100A6 mRNA and protein, while DPN, an ERβ agonist, had no obvious effect on S100A6 levels (Figure 8a,b). S100A6 immunofluorescence was also increased by estrogen or PPT treatment, which was blocked by ICI (Figure 8c). In ovariectomized ERα-knockout (ERαKO) mice, estrogen had no obvious effect on the levels of S100A6 mRNA and protein (Figure 8d,e). S100A6 immunofluorescence was also not induced by estrogen in ERαKO mice (Figure 8f). These data reveal that estrogen induces S100A6 expression through ERα.

## 4. Discussion

**Figure 9:**
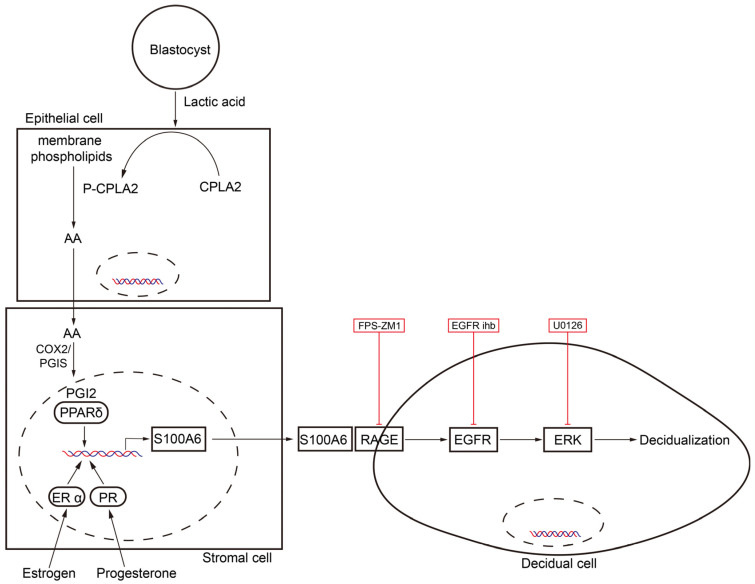
Graphic summary of the regulation and action of S100A6 during mouse implantation and decidualization. Blastocyst-derived lactate regulates S100A6 expression through the cPLA2/AA/COX2/PGI2/PPAR δ pathway. S100A6 regulates decidualization via the RAGE/EGFR/ERK pathway.

In our study, S100A6 is specifically expressed in mouse subluminal stromal cells from days 4 to 8 of pregnancy, suggesting that S100A6 may participate in implantation and decidualization. Blastocyst-derived lactic acid induces AA secretion by activating luminal epithelial p-cPLA2. Our data also show that the epithelial AA induces stromal S100A6 expression through the COX2/PGI2/PPAR δ pathway. Moreover, S100A6/RAGE signaling regulates decidualization via EGFR/ERK1/2 (Figure 9).

In our study, S100A6 signals first appear in the subluminal stroma on day 3 of pregnancy, as detected by immunofluorescence and in situ hybridization. Progesterone levels from the newly formed ovarian corpora lutea are also increasing during this time [36]. Previous studies showed that progesterone induces S100 gene expression in rat adipose-derived stem cells [37], as shown in our results. On the morning of the fourth day, a small surge of ovarian estrogen is essential for the formation of competent blastocysts and a receptive endometrium [38]. We observed an increase in S100A6 expression in the subluminal stroma on day 4 of pregnancy, which led us to speculate that estrogen also regulates S100A6 expression. An inductive relationship between estrogen and S100A6 expression was shown during the follicular phase of the estrous cycle in adult female rats [39]. Our results are consistent with these findings, as we observed that S100A6 expression is regulated by progesterone and estrogen in a PR- and ER-dependent manner.

Evidence suggests that a growing body of S100 protein family members are important for implantation and decidualization. S100A8 can affect embryonic development. S100A8 levels in the maternal decidual zone are related to vasculogenesis [40]. S100A4 participates in endometrial receptivity and decidualization [41]. S100A10 is increased in implantation and decidualization, and a reduction in S100A10 expression leads to repeated implantation failure [42]. Taken together, our results showing that S100A6 is increased in the decidual zone from days 5 to 8 confirm that S100A6 is also involved in decidualization. Our in vitro experiments involving the knockdown or overexpression of S100A6 further verified that S100A6 can affect the process of decidualization.

S100A6 can interact with RAGE [43] and integrin β1 [44]. The RAGE expression pattern in the mouse uterus is similar to that of S100A6 expression. It is possible that RAGE may act as a cell surface receptor for S100A6 to transduce extracellular effects. RAGE is considered to be a pattern-recognition receptor [45]. RAGE contributes and mediates sterile inflammatory responses by binding a range of endogenous damage-associated molecular-pattern molecules (DAMPs), such as HMGB1, S100s and DNA [46,47]. HMGB1 can induce vitro decidualization by luminal epithelium-derived HB-EGF [23]. ATP is an important member of DAMPs and can also promote decidualization in mice and humans [31]. Therefore, we propose that S100A6 may bind to RAGE as part of the process of implantation and decidualization. In HaCaT keratinocytes, S100A6 can activate ERK1/2 and STAT3 via EGFR to promote cell proliferation [35]. p-ERK1/2 is strongly detected at implantation sites and is essential to mouse and human decidualization [48]. The EGFR/ERK1/2 pathway is also involved in the process of decidualization [49]. Our data suggest that S100A6 regulates mouse decidualization via the RAGE/EGFR/ERK1/2 pathway.

Based on the comparison between delayed implantation and activation implantation, between day 4 of pregnancy and pseudopregnancy, and between implantation and inter-implantation sites, blastocysts may be involved in regulating S100A6 expression. In our study, blastocyst-derived lactic acid can stimulate S100A6 expression through luminal epithelial and stromal cell crosstalk. However, active blastocysts can also secrete proinflammatory factors, including S100A9, TNF and ATP [50]. Whether these molecules can regulate S100A6 expression remains to be defined. Multiple transcription factors are possibly involved in activating the S100A6 gene promoter, including NF-κB, USF and Nrf [51,52,53]. Based on predictions using the Animal TFDB database, a PPAR δ binding site can be found in the promoter of S100A6. PGI2 is the major member of the prostaglandin family that acts in the initiation of implantation and decidualization via PPAR δ [54,55]. In the mouse endometrium, COX2, PGI2 and PPAR δ are highly detected at the implantation site and the decidualization zone [56,57]. These expression and location coincided with those of S100A6. Our data suggest that AA may induce S100A6 expression through the COX2/PGI2/PPAR δ pathway in stromal cells. Therefore, PGs may regulate the expression of S100A6 via the PPAR δ pathway. cPLA2α is expressed in the human and mouse endometrium, which plays an important role in implantation and decidualization by catalyzing arachidonic acid to regulate prostaglandin synthesis [9,10]. Similarly, our data suggest that lactic acid stimulates the expression of AA and P-cPLA2 in epithelial cells. Therefore, our data show that blastocyst-derived lactic acid could induce stromal S100A6 expression through the AA-COX2-PGI2-PPAR δ pathway.

The inflammatory response is crucial for rodent and human implantation and decidualization [3]. There are many inflammatory molecules related to implantation and decidualization, including LIF, PGE2, COX2, PGI2, IL-6, IL8 and TNF [58]. Local injury by endometrial biopsy can increase the implantation rate by upregulating the inflammation response [59]. Women consuming anti-inflammatory drugs during early pregnancy have an increased rate of miscarriage due to defective implantation [60]. In sterile liver injury, S100A6, as a danger signal, activates the inflammatory response [18]. Among the S100A family, S100A8 plays an important role during inflammatory processes [61]. In inflammatory ocular diseases, S100A9 and S100A8 are upregulated, which may activate the innate immune system and alter immune tolerance [62]. In our study, S100A6 is highly expressed in subluminal stromal cells from days 5 to 8 of pregnancy. S100A6 knockdown compromises the process of decidualization, while S100A6 overexpression promotes in vitro decidualization. Thus, S100A6 could act as an inflammatory mediator to play a significant role during implantation and decidualization.

## 5. Conclusions

Overall, S100A6, as an inflammatory mediator, is important for mouse decidualization. Blastocyst-derived lactic acid may also be essential in this process, inducing stromal S100A6 expression via luminal epithelial AA secretion.

## Data Availability

The research data of the current study are included in this manuscript in the attached file.

## Abbreviations

AA	Arachidonic acid
COX2	Cyclooxygenase 2
EGFR	Epidermal growth-factor receptor
HMGB1	High-mobility-group box 1
MET	Mesenchymal-to-epithelial transition
PGI2	Prostacyclin
PPAR δ	Peroxisome proliferator-activated receptor
Prl8a2	Prolactin family 8, subfamily A, member 2
RAGE	Receptor for advanced glycation end products
